# Comparison of Antioxidant Capacity and Network Pharmacology of Phloretin and Phlorizin against Neuroinflammation in Traumatic Brain Injury

**DOI:** 10.3390/molecules28030919

**Published:** 2023-01-17

**Authors:** Kubra Kizil Ongay, Daniel Granato, George E. Barreto

**Affiliations:** Department of Biological Sciences, Faculty of Science and Engineering, University of Limerick, V94 T9PX Limerick, Ireland

**Keywords:** traumatic brain injury, dihydrochalcones, bioactive compounds, estrogen receptors, antioxidants, neuroinflammation

## Abstract

Neuroinflammation is a hallmark of traumatic brain injury (TBI)’s acute and chronic phases. Despite the medical and scientific advances in recent years, there is still no effective treatment that mitigates the oxidative and inflammatory damage that affects neurons and glial cells. Therefore, searching for compounds with a broader spectrum of action that can regulate various inflammatory signaling pathways is of clinical interest. In this study, we determined not only the in vitro antioxidant capacity of apple pomace phenolics, namely, phlorizin and its metabolite, phloretin, but we also hypothesize that the use of these bioactive molecules may have potential use in TBI. We explored the antioxidant effects of both compounds in vitro (DPPH, iron-reducing capacity (IRC), and Folin–Ciocalteu reducing capacity (FCRC)), and using network pharmacology, we investigated the proteins involved in their protective effects in TBI. Our results showed that the antioxidant properties of phloretin were superior to those of phlorizin in the DPPH (12.95 vs. 3.52 mg ascorbic acid equivalent (AAE)/L), FCRC (86.73 vs. 73.69 mg gallic acid equivalent (GAE)/L), and iron-reducing capacity (1.15 vs. 0.88 mg GAE/L) assays. Next, we examined the molecular signature of both compounds and found 11 proteins in common to be regulated by them and involved in TBI. Meta-analysis and GO functional enrichment demonstrated their implication in matrix metalloproteinases, p53 signaling, and cell secretion/transport. Using MCODE and Pearson’s correlation analysis, a subcluster was generated. We identified ESR1 (estrogen receptor alpha) as a critical cellular hub being regulated by both compounds and with potential therapeutic use in TBI. In conclusion, our study suggests that because of their vast antioxidant effects, probably acting on estrogen receptors, phloretin and phlorizin may be repurposed for TBI treatment due to their ease of obtaining and low cost.

## 1. Introduction

Traumatic brain injury (TBI) is a significant risk for all people worldwide, regardless of age, gender, and geography, and is the leading cause of accidental death and disability [1,2]. TBI mainly occurs due to traffic accidents, falls, and sports accidents [3,4]. There is no complete treatment approach for TBI yet. Although the treatment methods used to prevent the progression of brain damage are quite expensive in low- and middle-income countries, failure to make an accurate and timely diagnosis and poor patient outcomes cause high rates of death and disability in these countries [5]. TBI, divided into primary and secondary brain damage according to the severity of the injury [6], can be defined as the dysfunctions of brain cells after the damage [7]. Although the pathophysiology of TBI is not yet fully known, the most critical condition is inflammation in the brain [8,9]. With the understanding of the biological process of the disease, the development of appropriate treatment methods will open up, and death and disability can be prevented with advantages such as early diagnosis and prevention of the progression of the disease.

Several mechanisms are involved in the pathophysiology of TBI. One of them, inflammation, occurs in secondary damage following the primary injury, becomes chronic when left untreated, and causes various degenerative diseases in the future [10]. Although some anti-inflammatory drugs used in treating TBI today have shown benefits in clinical trials, none provide a complete recovery [11,12].

One of the main hallmarks of TBI pathology is hypogonadism. Some patients present hormonal disorders such as deficiencies in sex hormones after TBI [13,14,15,16], and a similar outcome is also observed in TBI animal models [17]. These deficiencies in estrogen and testosterone are known as hypogonadism [16]. Estrogens and testosterone both have vast protective effects in the central nervous system (CNS) against chronic inflammation by alleviating the activation of glial cells (namely astrocytes and microglia) [18,19,20,21,22,23,24,25,26] and inhibiting the secretion of inflammatory cytokines [12,27,28,29]. Hence, the neuroprotective effects of both hormones may be a novel therapeutic approach that could be used in treating TBI [12].

**Figure 1:**
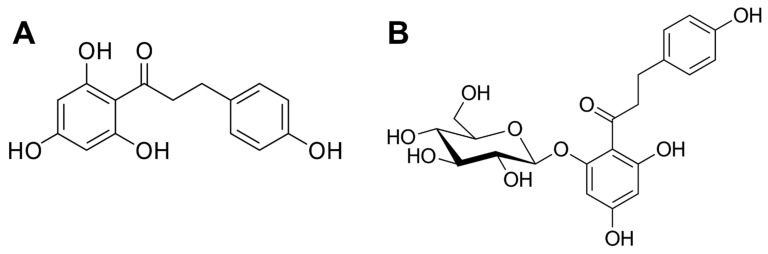
Chemical structures of phloretin (**A**) and phlorizin (**B**).

In recent years, many attempts to discover new and more specific pharmacological therapies to alleviate TBI damage have been explored [18,30,31]. Causes of secondary brain injury, such as inflammation and neuronal death, can also be treated with natural compounds [32,33,34,35] obtained from plants; they are used in treating many diseases due to their potent antioxidant effects [33,36,37] neutralizing reactive oxygen and nitrogen species (ROS/RNS), hence mitigating inflammation. In the case of TBI, they may be used for repurposing by reducing edema and exerting protection of the blood–brain barrier [38]. Recently, our group became interested in exploring the neuroprotective properties of phlorizin and its derivative, phloretin (Figure 1) [39,40], two compounds that are primarily found in apples and their industrial by-product, apple pomace. Interest in the biological activities of these compounds is associated mainly with their antioxidant, anti-inflammatory, and estrogenic properties. However, their molecular mechanism of protection in TBI is not comprehensively unveiled. Therefore, considering the gap in the literature and the need to study the mechanism of action of natural compounds, in this study, we determined not only the in vitro antioxidant capacity of apple pomace phenolics, namely phlorizin and its metabolite, phloretin, but we also hypothesize that the use of these bioactive molecules may have potential use in TBI.

## 2. Results

### 2.1. Antioxidant Activities of Phlorizin and Phloretin

**Figure 2:**
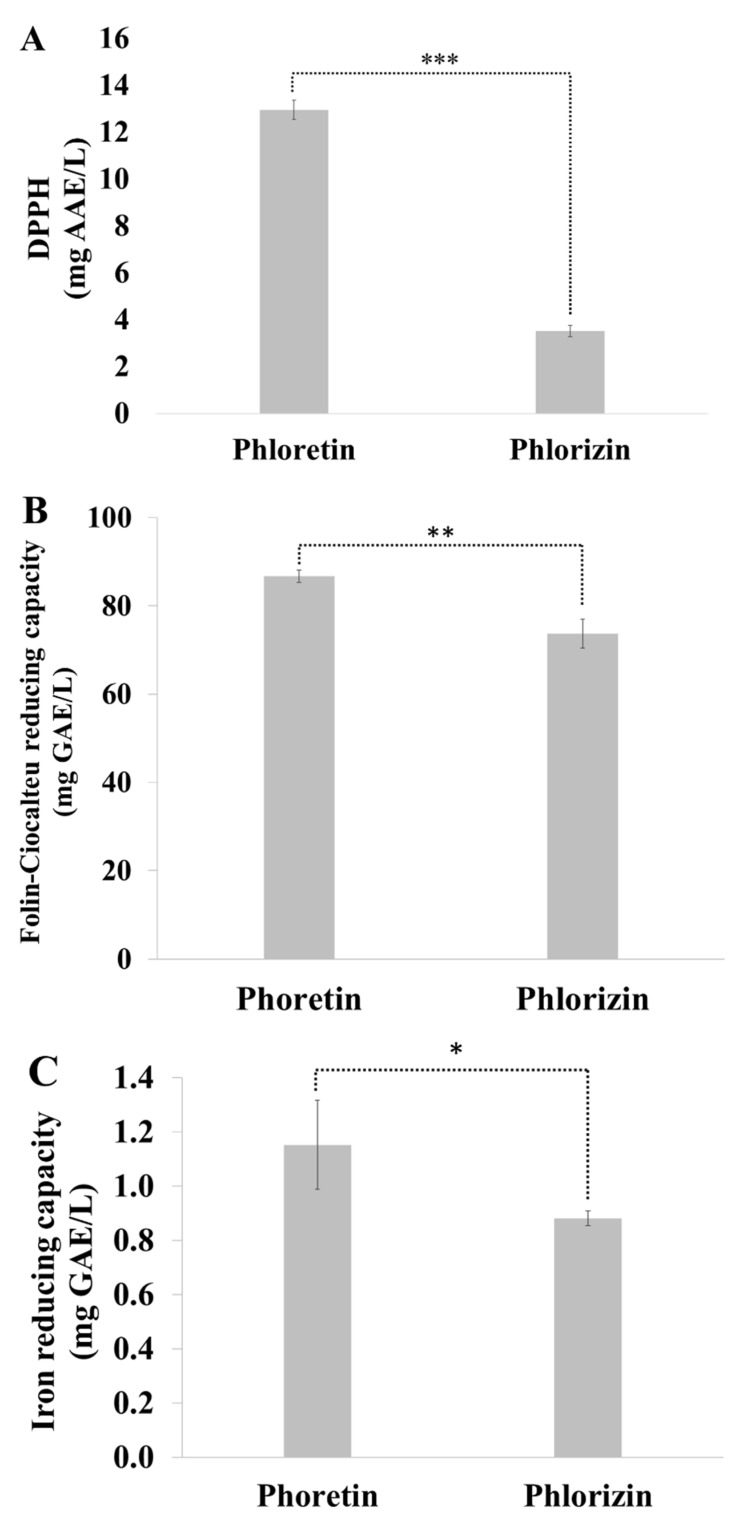
Antioxidant capacity of phloretin and phlorizin tested at 0.5 mmol/L using different in vitro assays: free-radical scavenging activity in relation to DPPH (**A**), Folin–Ciocalteu reducing capacity (**B**), and iron-reducing capacity (**C**). ***p < 0.0001, ** *p* < 0.001, * *p* < 0.05.

To investigate possible antioxidant properties that might explain the anti-inflammatory action of phlorizin and phloretin, we initially tested these compounds in three different methods. The DPPH assay results showed that phloretin (12.95 mg AAE/L) had a 3.7-fold higher (*p* < 0.0001) free-radical scavenging activity than phlorizin (3.52 mg AAE/L). Similarly, phloretin showed higher reducing potential, as formally checked by two distinct assays. For the FCRC, phloretin had a 15% higher (*p* < 0.001) mean value than phlorizin (86.73 and 73.69 mg GAE/L, respectively), while for the IRC, phloretin had a 23.5% higher (*p* < 0.05) mean value than phlorizin (1.15 and 0.88 mg GAE/L, respectively) (Figure 2A–C).

### 2.2. Potential Molecular Targets of Phlorizin and Phloretin in TBI Pathology

**Figure 3:**
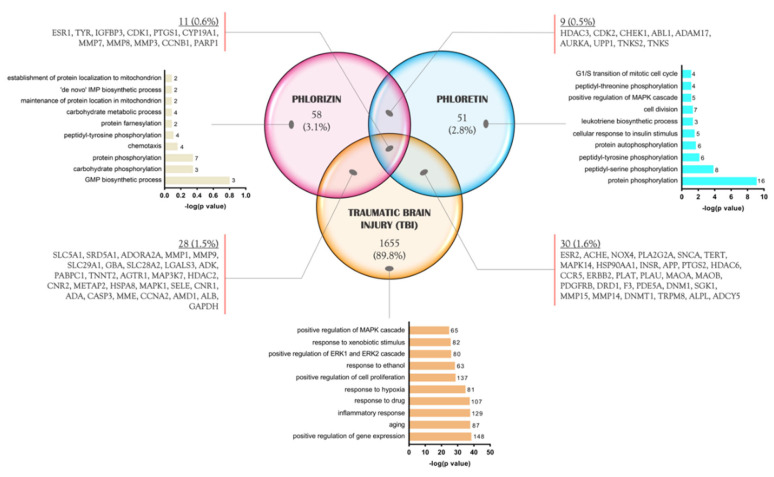
Venn diagram of common proteins between phlorizin, phloretin, and TBI. In the diagram prepared using the lists from SEA, SwissTargetPrediction, CTD, and GeneCards/Malacards database, it is seen that approximately 0.6% (11) of the proteins are shared between the three groups.

To better understand the antioxidant effects of each compound seen in Figure 1 and to predict how they might reduce brain damage by regulating different signaling pathways after a TBI event, we explored and determined those proteins that are unique and potential targets in three lists (phlorizin, phloretin, and TBI). Approximately 58 (3.1%) proteins are regulated by phlorizin, whose biological processes include post-translational mechanisms such as phosphorylation and farnesylation, metabolism of metabolic substrates, and nucleotide synthesis (Figure 3). On the other hand, 51 (2.8%) of the total proteins modulated by phloretin participate in the phosphorylation of amino acids and proteins, regulation of cellular survival pathways such as MAPK, cell proliferation and cell cycle, and cellular response to insulin.

Interestingly, the biological category that appears in the phloretin list, and not in phlorizin’s, is cell division control processes. In contrast, in phlorizin, the localization of proteins in the mitochondria is the most regulated. Finally, considering only those proteins regulated on the TBI list, 1655 (89.8%) are involved in the inflammatory response, aging, hypoxia response, and mediation of proliferative processes and pathways such as ERK1/ERK2 and MAPK (Figure 3).

When crossing the three lists to identify those proteins in common, 9 (0.5%) were unique among phlorizin/phloretin, 28 (1.5%) were in the phlorizin/TBI lists, and 30 (1.6%) were in common among phloretin/TBI. Remarkably, 11 (0.6%) proteins (ESR1 (estrogen receptor alpha), TYR (tyrosinase), IGFBP3 (insulin-like growth factor binding protein 3), CDK1 (cyclin-dependent kinase 1), PTGS1 (prostaglandin-endoperoxide synthase 1), CYP19A1 (cytochrome P450 Family 19 subfamily A member 1), MMP7 (matrix metallopeptidase 7), MMP8 (matrix metallopeptidase 8), MMP3 (matrix metallopeptidase 3), CCNB1 (cyclin B1), and PARP1 (Poly(ADP-Ribose) polymerase 1)) were identified as being shared among the three analyzed lists and will be explored in more detail (Figure 3).

### 2.3. Enrichment Analysis and Meta-Analysis of Common Proteins

**Figure 4:**
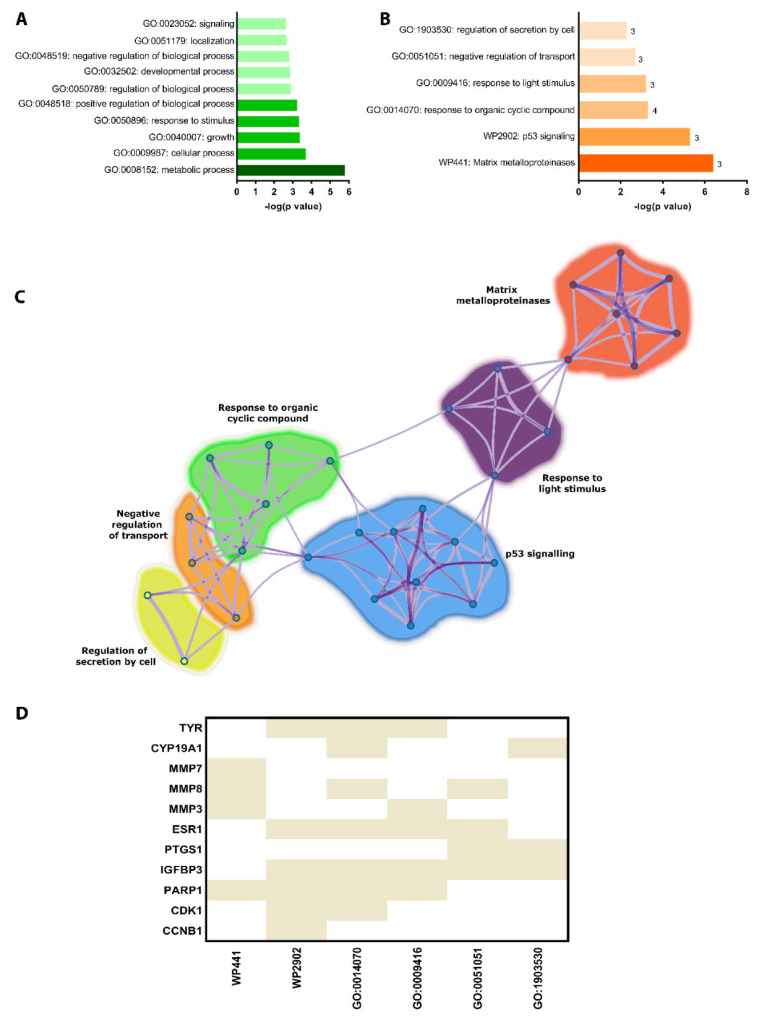
Functional enrichment and meta-analysis of 11 proteins in common among the three lists. Top-level biological processes (**A**). Enriched terms across the 11 common proteins in the three lists (**B**). Inter-relationship between the enriched categories (**C**). Heatmap showing the number of times each protein appears in enriched terms (**D**).

In an initial analysis, we hypothesized which biological processes these proteins might be involved in and how they could influence the inflammatory mechanism in TBI. To reach this goal, we generated a list of the top biological processes that include, amongst others, metabolic process (GO:0008152) [−log(*p*-value): 5.77] as the most regulated (Figure 4A). This category includes anabolic and catabolic processes of energy substrates and the biotransformation and metabolism of small molecules and large macromolecular complexes with DNA and other proteins. This is followed by GO:0009987 cellular process [−log(*p*-value): 3.70], GO:00400007 growth [−log(*p*-value): 3.36], GO:0050896 response to stimulus [−log(*p*-value): 3.33], GO:0048518 positive regulation of biological process [−log(*p*-value): 3.22], GO:0050789 regulation of biological process [−log(*p*-value): 2.89], GO:0032502 developmental process [−log(*p*-value): 2.85], GO:0032502 negative regulation of biological process [−log(*p*-value): 2.80], GO:0051179 localization [−log(*p*-value): 2.66], and GO:0023052 signaling [−log(*p*-value): 2.62].

Next, we explored all statistically enriched terms, where *p*-values and enrichment factors were used for filtering. Hierarchically and clustered terms were GO:1903530 regulation of secretion by cell [−log(*p*-value): 2.31], GO:0051051 negative regulation of transport [−log(*p*-value): 2.66], GO:0009416 response to light stimulus [−log(*p*-value): 3.24], GO:0014070 response to organic cyclic compounds [−log(*p*-value): 3.34], WP29602 p53 signaling [−log(*p*-value): 5.28], and WP441 matrix metalloproteinases [−log(*p*-value): 6.39] (Figure 4B).

To better understand the relationship between the terms observed in Figure 3B, a subgroup of the most enriched terms was generated to determine how they interact and to identify those with the greatest significance within the network (Figure 4C). Therefore, we classified those proteins that appear one or more times in these enriched terms to highlight the importance of each one in modulating various signaling pathways. These results show that IGFBP3 appears five times, while ESR1 and PARP1 appear four times each (Figure 4D).

### 2.4. PPI Analysis of Target Proteins

**Figure 5:**
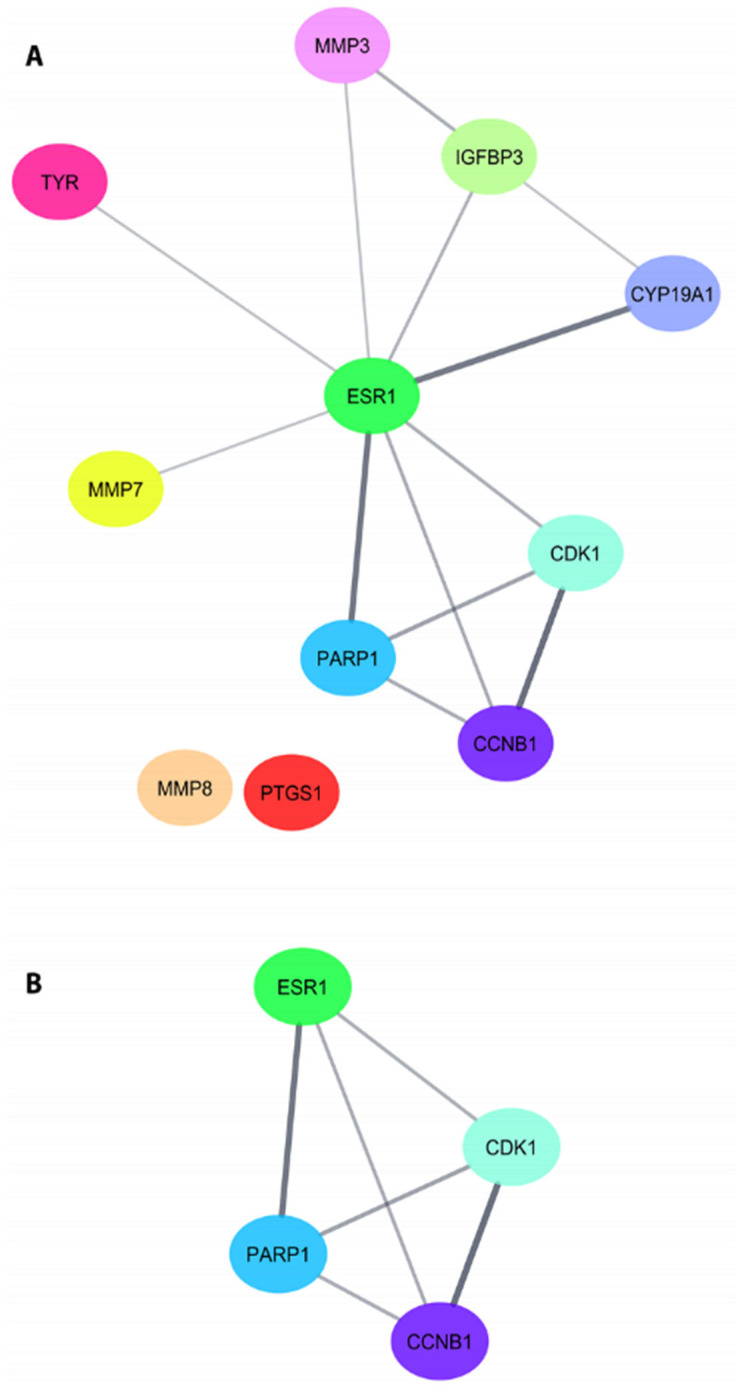
Protein–protein interaction network formed by common proteins between phlorizin, phloretin, TBI (**A**), and MCODE components (**B**), showing ESR1 as the primary cellular hub protein.

Protein–protein interaction analysis of 11 proteins common among phlorizin, phloretin, and TBI was performed using Cytoscape. In this analysis, it was observed that MMP8 and PTGS1 proteins are singletons, and the most robust interactions are between PARP1-ESR1 (score value: 0.95), CYP19A1-ESR1 (score value: 0.922), and CCNB1-CDK1 (score value: 0.999) (Figure 5A). To determine the possible cluster in this network, the MCODE module, which considers the values of betweenness centrality, closeness centrality, and degree, was used, and a cluster consisting of four proteins (Score: 4.0), ESR1, PARP1, CDK1, and CCNB1, was reached (Figure 5B).

To classify proteins, aiming to identify those essential for the flow of information across the network, we applied criteria such as degree, betweenness, and closeness centrality. When the values specified in detail for the 11 proteins in Table 1 are compared, it is seen that the ESR1 protein has the highest values in the network with a degree of 8, betweenness centrality of 0.803571429, and closeness centrality of 1.0, making this protein an essential cellular hub within the network.

**Figure 6:**
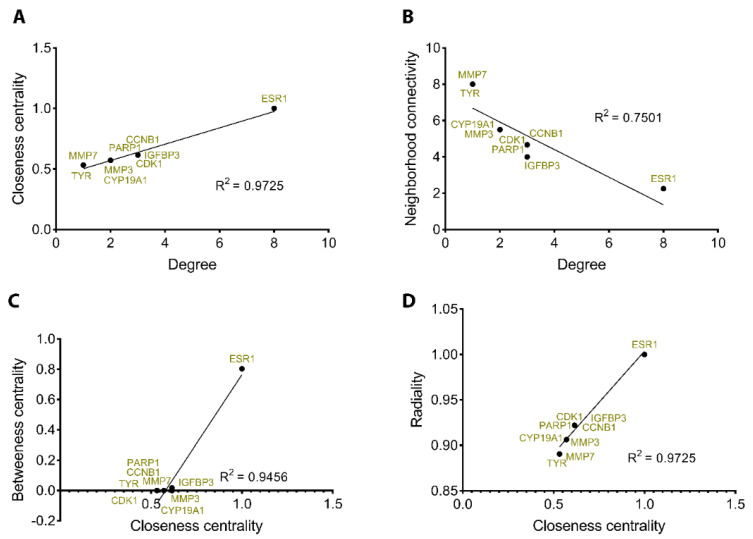
Validation of cellular hubs using Pearson’s correlation. ESR1 is a key molecular hub, positioning ahead of the other clustered proteins when closeness vs. degree (**A**), neighborhood vs. degree (**B**), closeness vs. betweenness (**C**), and closeness vs. radiality (**D**) are assessed in terms of functional correlation.

To confirm and validate the previous results, we performed an additional correlation analysis between degree, closeness, betweenness, neighborhood connectivity, and radiality to identify the highly regulated cellular targets (hubs) in our PPI network (Figure 5). The positive interaction between closeness and degree with an R^2^ = 0.9725 shows ESR1 as being the protein with the highest scores (Figure 6A). Similarly, ESR1 appears again ahead of the other proteins when neighborhood connectivity, closeness and betweenness, and radiality interact, showing a significant positive correlation between them (Figure 6B–D).

## 3. Discussion

A critical aspect of TBI pathology is hypogonadism, a clinical symptom that can contribute to the progression of the disease and worsen its prognosis. Decreased hormone levels also significantly impact metabolism and cell death in post-trauma periods, which makes us hypothesize that restoring hormonal control of brain functions with exogenous hormones can serve as a pharmacological treatment to reduce the mortality of neurons and other brain cells, such as astrocytes and microglia. Although both compounds (phlorizin and phloretin) investigated here have demonstrated decisive anti-inflammatory actions [39,41,42,43,44], their possible molecular actions have not been studied in TBI. Our goal was to decipher which proteins regulated by phlorizin and phloretin, two natural compounds with estrogenic activity, can potentially regulate pro-oxidative and inflammatory mechanisms, which are the main hallmarks of TBI.

Phenolic compounds are known for donating electrons or hydrogen atoms to stabilize free radicals and other nonradical reactive species, such as hydrogen peroxide [45]. Phloretin has a greater antioxidant capacity than its precursor, phlorizin, using three assays: inhibition of DPPH radical, FCRC, and IRC. These methods encompass single electron transfer (FCRC and IRC) and a mixture of hydrogen atom transfer and single electron transfer (DPPH). Unlike phloretin, phlorizin contains a beta-D-glucopyranosyl residue at position 2’ via a glycosidic linkage in its structure. While this bond is not stable, it is also prone to hydrolysis, which may somewhat explain its lower scavenging activity. In fact, the glycosylation of phenolic compounds tends to decrease the extent of deprotonation, thus decreasing the quenching of free radicals [46]. In vivo intestinal β-glucosidase inhibitors convert phlorizin to its aglucon form, phloretin, enhancing its free-radical scavenging and reducing capacities [47]. Because of such beneficial properties, antioxidants are preferred as natural methods for mitigating the harmful effects of various diseases, particularly TBI, which is characterized by massive death of neurons and astrocytes, leading to severe motor and sensory consequences.

One of the key proteins and the central cellular hub involved in the molecular effects of phloretin and phlorizin is ESR1. Here we observed that one of their main targets is the estrogen receptor alpha (ERα), which has been reported to have broad neuroprotective effects against a wide variety of neurological diseases [48,49,50,51,52], including TBI [27,53]. By interacting with p53 and metalloproteinases such as MMP3 and MMP7 (Figure 5), ERα can modulate apoptotic signals and cell survival processes. In fact, administration of 17-β-estradiol (180 μg/mL in capsules), its primary substrate, mitigates apoptotic cell death by inhibiting caspase-3 activation in areas close to the injury site in ovariectomized animals after traumatic brain injury [54]. Both ERα mRNA and protein upregulation by 17-β-estradiol are thought to be involved in its antiapoptotic mechanisms, which was confirmed after using inhibitors to block this receptor; its actions on caspase-3 cleavage were completely abolished [54,55]. Moreover, using DPN, an ERα specific agonist, lowers water brain content and improves neurological scores in TBI animals [56]. Acting on ERα 17-β-estradiol regulates metalloproteinases, particularly MMP3, and alleviates tight junction dysfunction, preserving BBB in vitro in endothelial cells exposed to oxygen–glucose deprivation [57]. p53 is induced in the cortex, thalamus, and hippocampus after TBI [58], suggesting an increase in apoptosis. Interestingly, estradiol interacts with p53 through its ligase, Mdm2 (E3 ubiquitin–protein ligase), which causes ubiquitination of p53, thus reducing the activation of apoptosis. Although estradiol induces p53 acetylation, which further reduces its activation [59], it is yet to be explored whether these effects of estradiol on p53 are mediated by ERα or the other isoforms (e.g., ERβ). Little is known whether both phlorizin and phloretin can induce antioxidant effects via ERα regulation, with just one interaction study confirming phloretin estrogenicity [60], with particular emphasis on ERα [61].

## 4. Materials and Methods

### 4.1. Identification of Proteins Regulated by Phlorizin and Phloretin in TBI Pathology

We initially explored those proteins that have been reported to be regulated by the compounds under the current study that may have an involvement in the pathology of TBI. For that, we used the simplified molecular input line entry system (SMILES) from PubChem (https://pubchem.ncbi.nlm.nih.gov accessed on 25 September 2022) for each compound in the similarity ensemble approach (SEA; https://sea.bkslab.org) (accessed on 25 September 2022) and SwissTargetPrediction (http://swisstargetprediction.ch) (accessed on 25 September 2022) databases to list the proteins that were regulated by phlorizin and phloretin. Searches were carried out using the terms “phlorizin” and “phloretin”. After this step, we eliminated the duplicates to maintain a single list of proteins in each category. A total of 371 proteins were identified as being regulated by phlorizin and phloretin.

To build up TBI lists, a total of 1742 related proteins were retrieved in the GeneCards/Malacards (https://www.genecards.org) database (accessed on 25 September 2022) and CTD (Comparative Toxigenomics database) search using the terms “traumatic brain injury” and “brain trauma” for the analysis of proteins associated with TBI pathology.

### 4.2. Protein–Protein Interaction (PPI) Network and Functional Enrichment Analysis

First, the shared protein list among phloretin, phlorizin, and TBI was created by comparing these three lists in a Venn diagram. The protein–protein interactions were analyzed in Cytoscape version 3.9.0 (https://cytoscape.org, accessed on 25 September 2022) [62]. Then, the protein query module STRING and ”Analyse Network” tool with the confidence (score) cut-off of 0.4 and the maximum additional interactors 0 were used to calculate betweenness centrality (the shortest path between the proteins), closeness centrality (the distances of were used to calculate the proteins to each other), and degree (the number of interactions a protein makes in the network) for each protein in the network.

The MCODE (molecular complex detection) module was used to determine the most crucial cluster in the network, with a cut-off value of 2, a node score cut-off value of 0.2, a K-core of 2, and a maximum depth of 100. The MCODE module creates the appropriate cluster by considering the betweenness centrality, closeness centrality, and degree values of the proteins. Pearson’s correlation coefficients were calculated using Cytoscape to compare these parameters.

For pathway and GO biological processes functional enrichment analysis, we used KEGG (Kyoto Encyclopaedia of genes and genomes), DAVID v2022q3 (Database for Annotation, Visualization and Integrated Discovery), and PANTHER version 17.0 pathways.

### 4.3. Meta-Analysis of Common Proteins among Phlorizin, Phloretin, and TBI

Using Metascape [63], we analyzed the proteins found significantly among the lists using the BioGrid, Omnipath, and STRING databases. The generated protein–protein network included at least one interaction where they share a functionality. Those proteins with a significant *p*-value (>0.05) and false discovery ratio (FDR) were included for further analysis.

### 4.4. Antioxidant Capacity In Vitro

The antioxidant capacity of phloretin and phlorizin was assayed using three different protocols: free-radical scavenging activity in relation to DPPH, Folin–Ciocalteu reducing capacity (FCRC), and iron-reducing capacity (IRC). First, the compounds were diluted with methanol at a molar concentration of 0.5 mmol/L to compare the results on a molar basis. The methods followed the experimental conditions adopted and validated in our previous studies [64,65]. For the DPPH assay, a 0.10 mmol/L methanolic solution of DPPH was prepared, and the absorbance decay was monitored at 517 nm after 30 min reaction. The results were expressed as mg ascorbic acid equivalents/L (mg AAE/L). For the FCRC assay, Folin–Ciocalteu phenol reagent (diluted 1:3 *v*/*v* in water) was added to the diluted samples, and the reaction was completed using a 10 g/100 mL solution of Na_2_CO_3_, and the absorbance was read at 725 nm after 60 min of action. The results were expressed as mg of gallic acid equivalents/L (mg GAE/L). For the IRC assay, the colorimetric assay that employs FeCl_3_.6H_2_O and K_3_[Fe(CN)_6_] as chromophores at 0.5 mmol/L was used, and the absorbance was read at 725 nm after 15 min of reaction. The results were expressed as mg GAE/L. For all assays, the coefficient of determination (R^2^) in the linearity test (i.e., analytical curves) was higher than 0.990.

### 4.5. Statistical Analysis

Results were expressed as the mean values followed by the standard deviation (n = 3 replicates per assay). To compare the antioxidant capacity between phloretin and phlorizin, a Student’s *t* test for independent samples was applied, taking on *p* < 0.05 to highlight differences. The software TIBCO Statistica v. 13.3 (TIBCO Software Inc., Palo Alto, CA, USA) was used in the experiments.

## 5. Conclusions and Future Perspectives

In this study, phlorizin and phloretin were compared in terms of their in vitro antioxidant potential, and we found out that phloretin showed higher single-electron-transfer and hydrogen-atom-transfer abilities compared to phlorizin, using three distinct assays. Because of their antioxidant potential, these compounds have promising protective effects against TBI pathology. Using computational and in vitro assays, the potential therapeutic targets of phlorizin and phloretin in TBI pathology were examined. It was seen that one of the proteins highly regulated by these natural antioxidants is ESR1.

Estrogen is of great importance for treating hypogonadism, which is defined as a reduction in sex hormones. To prevent the immediate and long-term effects of these deficiencies in patients, further research should be conducted on phlorizin and phloretin as a new potential treatment method in post-TBI hypogonadism. The high amount of phlorizin and phloretin found in apples, which can be found worldwide, allows easily accessible treatments to be implemented in public health practices.

## Figures and Tables

**Table 1:** Parameters used to evaluate 11 common proteins between phlorizin, phloretin, and TBI in protein–protein interaction analysis by Cytoscape.

Protein	Degree	Betweenness Centrality	Closeness Centrality	Neighborhood Connectivity	Radiality
ESR1	8	0.803571429	1	2.25	1
IGFBP3	3	0.017857143	0.615384615	4	0.921875
CCNB1	3	0	0.615384615	4.666666667	0.921875
PARP1	3	0	0.615384615	4.666666667	0.921875
CDK1	3	0	0.615384615	4.666666667	0.921875
MMP3	2	0	0.571428571	5.5	0.90625
CYP19A1	2	0	0.571428571	5.5	0.90625
TYR	1	0	0.533333333	8	0.890625
MMP7	1	0	0.533333333	8	0.890625
MMP8	0	0	0	0	Infinity
PTGS1	0	0	0	0	Infinity

## Data Availability

Not applicable.

## Sample Availability

Samples of the compounds are commercially available.
